# Insights into the Functional Roles of N-Terminal and C-Terminal Domains of *Helicobacter pylori* DprA

**DOI:** 10.1371/journal.pone.0131116

**Published:** 2015-07-02

**Authors:** Gajendradhar R. Dwivedi, Kolluru D. Srikanth, Praveen Anand, Javed Naikoo, N. S. Srilatha, Desirazu N. Rao

**Affiliations:** Department of Biochemistry, Indian Institute of Science, Bangalore, India; Liverpool University, UNITED KINGDOM

## Abstract

DNA processing protein A (DprA) plays a crucial role in the process of natural transformation. This is accomplished through binding and subsequent protection of incoming foreign DNA during the process of internalization. DprA along with Single stranded DNA binding protein A (SsbA) acts as an accessory factor for RecA mediated DNA strand exchange. *H*. *pylori* DprA (HpDprA) is divided into an N-terminal domain and a C- terminal domain. In the present study, individual domains of HpDprA have been characterized for their ability to bind single stranded (ssDNA) and double stranded DNA (dsDNA). Oligomeric studies revealed that HpDprA possesses two sites for dimerization which enables HpDprA to form large and tightly packed complexes with ss and dsDNA. While the N-terminal domain was found to be sufficient for binding with ss or ds DNA, C-terminal domain has an important role in the assembly of poly-nucleoprotein complex. Using site directed mutagenesis approach, we show that a pocket comprising positively charged amino acids in the N-terminal domain has an important role in the binding of ss and dsDNA. Together, a functional cross talk between the two domains of HpDprA facilitating the binding and formation of higher order complex with DNA is discussed.

## Introduction

DprA, a recombination mediator protein, plays a crucial role in the natural transformation pathway favouring genetic diversity of the bacteria. DprA was first identified in *Haemophilus influenzae* in an experiment involving random mutagenesis [1]. In *H*. *Influenza*[1] and *Streptococcus pneumoniae* [2], knockout of DprA resulted in reduction of transformation efficiency with chromosomal DNA but not plasmid DNA. However, it was later observed that there are two alternative single strand annealing pathways for plasmid transformation with RecO and DprA potentially having redundant roles [3]. Disruption of *dprA* in *H*. *Pylori*[4,5], *Campylobacter jejuni*[6] and *Bacillus subtilis*[7] reduced transformation efficiency for both chromosomal DNA and plasmid DNA. A functional role for DprA was identified for DNA processing subsequent to its uptake in *S*. *pneumonia* [8].

DprA is a DNA binding protein, characterized by presence of a conserved DNA binding domain. The DNA binding domain adopts a Rossmann fold like topology spanning most of the protein. A Rossmann fold consists of alternating alpha helix and beta strands in the topological order of β-α-β-α-β[9]. All homologous DprA proteins characterized till date show that in addition to the prominent Rossmann fold domain they consist of one or more smaller domains. *Rhodopseudomonaspalustris* DprA (RpDprA) consists of two more domains besides the Rossmann fold domain i.e., N-terminal SAM (sterile alpha motif) domain and a C-terminal DML-1 like domain[10]. SAM is an evolutionary conserved protein binding domain that is involved in regulation of numerous developmental processes among diverse eukaryotes [11]. Similarly DML1 is considered to be a Z—DNA binding domain [12]. *Streptococcus pneumonia* DprA (SpDprA) consist of an N-terminal SAM domain other than Rossmann fold domain[13]. While the main function of Rossmann fold is to bind DNA, the supplementary domains are highly variable with respect to their sequence and function. For example, the SAM domain in *S*. *pneumoniae* plays a key role in switching off competence by directly interacting with ComE~P[14]. Initial biochemical studies with DprA from *S*. *pneumoniae* (SpDprA) reported that the protein binds internalized ssDNA tightly and protects it from various nucleases. It was also reported that SprDprA does not bind to dsDNA [15]. Our studies with HpDprA demonstrated the ability of the protein to bind both dsDNA and ssDNA with comparable affinities[16]. AFM (Atomic Force Microscopy) experiments demonstrated that *Bacillus subtilis* DprA (BsDprA) did not form any complex with dsDNA even at 200 fold higher concentration than required for formation of complex with ssDNA [17]. Hence, the homologous DprA may bind dsDNA but with very low affinity as compared to HpDprA. SpDprA was found to physically interact with RecA, alleviates SsbB (single stranded DNA binding protein involved in natural transformation) barrier and promotes its loading on ssDNA thus, favouring RecA catalyzed homology search and recombination [15]. However, it was later shown in *Bacillus subtilis* that both DprA as well as SsbA are required for strand exchange activity of RecA.ATP (RecA in ATP bound form) and SsbB act as accessory factors[18]. SpDprA exists as a dimer in solution and dimerization was observed to be mediated by Rossmann fold domain [13]. Dimerization of DprA was found to be important for it to interact stably with ssDNA.DprA dimer in complex with ssDNA has been shown to provide a platform for loading the first monomer of RecA on ssDNA and thus help in nucleation of RecA–ssDNA filament[19]. DprA in *H*. *pylori* was observed to modulate the restriction barrier by not only inhibiting the activity of restriction enzyme but also by stimulating activity of methyltransferase on DNA [16].

A Structure of HpDprA (from *H*. *pylori* strain 26695) in complex with ssDNA was investigated to understand the mechanistic detail of HpDprA interaction with DNA [10]. Crystal structure of HpDprA (strain 26695) revealed the presence of two domains i.e., N—terminal domain (Ser 12 to Asp 217) (designated as HpRF hereafter) and C- terminal domain (Met 226 to Ala 270) (designated as HpDML1 hereafter). Both these domains were linked together by 8 amino acid flexible linker (Tyr 218 –Glu 225)[10]. HpRF domain adopts a classical Rossmann fold like topology (nine α –helices alternating with nine β - strands) and the HpDML1 domain resembles *Drosophila melanogaster* misato like protein 1 (DML1 like) domain of *Rhodomonaspalustris* DprA[10]. HpDprA dimerization is mediated through hydrophobic inter- molecular interactions consisting of the residues from HpRF. Intermolecular hydrogen bonds on the reverse side of the hydrophobic core further enhance dimer formation [10]. HpRF consist of an extensively positive charged region (known as E1) neighbouring with a hydrophobic region (known as E2), which is considered mainly responsible for interaction of ssDNA with HpDprA [[10]. The crystal structure demonstrated a role for HpRF in dimer formation and in interaction with ssDNA. However, the role of HpDML1 is still elusive. Moreover, very little is known about the mechanistic detail of HpDprA interaction with dsDNA. In the current study, the role of HpRF and HpDML1 in formation of polymeric complex of HpDprA (from *H*. *pylori* strain J99) with DNA has been examined. Further, HpDprA interaction with dsDNA has been studied extensively. In the present study, the features gleaned from HpDprA crystal structure are utilized to discuss the possible role of HpRF and HpDML1 in polymerization of the protein on the DNA. HpRF binds ssDNA and dsDNA with lower affinity than full length protein, suggesting a role of HpDML1 in interaction with DNA. HpDprA was observed to possess two sites for self-interaction, one in HpRF and one in HpDML1 domain. The complex formed with truncated HpDprA was less stable compared to full length protein as analyzed by SPR. In conclusion, the functional cross talk between the domains of HpDprA during its interaction with DNA is discussed in this study.

## Materials and Methods

### Bacterial strains and plasmids

*H*. *pylori* J99 strain **(** *cagA* ^*+*^ *iceA1 vacAs1am1*) genomic DNA was obtained as a gift from New England Biolabs (Beverley, MA, USA). *Escherichia coli* strain DH5α **[**F' *end* A1 *hsd* R17 (r_k_^-^ m_k_^-^) *gln*V44 *thi*1 *rec*A1 *gyr*A (Nal^R^) *rel*A1 Δ (*lacI*ZYA–*arg*F) U169 *deo*R (Φ80d*lac* Δ (*lacZ*)M15}] was used as a host for preparation of plasmid DNA. *E*.*coli* BL21‐CodonPlus(DE3)‐RILstrain^*a*^(*E*.*coli* B F–*ompThsdS*(rB–mB–) *dcm*+ Tet^r^ *gal* λ (DE3) *endA*Hte*metA*::Tn5(Kan^r^) [*argUileYleuW*Cam^r^]) cells were obtained from Stratagene were used for expression and purification of HpDprA.

#### Reagents

Restriction endonucleases and T4 polynucleotide kinase were obtained from New England Biolabs. T4 DNA ligase and 1kb DNA ladder were obtained from Fermentas Life Sciences. PhusionDNA polymerase was obtained from Finnzymes. Coomassie brilliant blue R-250, proteinase K, Tris, heparin sepharose, protease inhibitor cocktail and IPTG were procured from Sigma chemical company (USA). Ni^+^-NTA agarose and Glutathione Sepharose were obtained from GE Healthcare (Sweden). [ᵞ^32^P]ATP (3500 Ci/mmol) was obtained from BRIT (India). All other reagents used were of analytical or ultrapure grade.

### Cloning, overexpression and purification of HpDML1 and HpRF

The nucleotide sequence corresponding to HpRF domain *hpdprA*_(1–636)_ and HpDML1 domain *hpdprA*_(637–801)_ of HpDprA were cloned in the pET28a vector under the control of T7 promoter. The primers used for the cloning are described in Table 1. Oligo 1, 2 were used for cloning of HpRF domain and oligos 3, 4 were used for cloning of HpDML1 domain. Both the proteins were overexpressed in *E*. *coli* BL21(DE3)-RIL harbouring the DNA construct pET28a-*hpdprA*_(1–636)_ and pET28a-*hpdprA*_(637–801)_. The recombinant bacteria were grown in LB broth with kanamycin selection (50 µg/ml) at 37˚C to A_600_ of 0.6. Expression of the proteins were induced by the addition of 0.5 mM IPTG, and the cultures were incubated for 4 h at 37˚C. Cells were collected by centrifugation, resuspended in buffer A [50 mM Tris–HCl (pH 7.4), 300 mM NaCl, 2 mM β-mercaptoenthanol, 10% (v/v) glycerol and 10 mM imidazole] and lysed by sonication. 1X protease inhibitor cocktail and 0.05% TritonX-100 was added to the cell suspension before sonication. The cell lysate was centrifuged at 16000 rpm for 1 h at 4˚C. The supernatant containing HpRF or HpDML1 protein was loaded onto a Ni-NTA column that had been previously equilibrated with buffer A. The column was washed with 30 column volumes of buffer A containing 30 mM imidazole. The protein was eluted with 10 ml of buffer A containing 300 mM imidazole. The eluate of Ni-NTA column was dialysed against 50 mM Tris buffer pH 7.4 containing 2 mM β mercaptoethanol, 10% glycerol and 75 mM NaCl. The dialyzed eluate of HpRF was loaded on a Heparin-Sepharose column. The column was washed with the same buffer and protein was eluted using a salt gradient of 0.1 M to 1 M. The purified protein was dialysed at 4°C against buffer A containing 200 mM NaCl. The dialyzed eluate of HpDML1 was loaded on a Superdex 200 HiLoad 16/600 column (GE Healthcare) pre-equlibrated with buffer containing 50 mM Tris (pH 7.6), 300 mM NaCl, and 2 mM β mercaptoethanol. The purity of the protein preparation was judged on SDS–PAGE with Coomassie brilliant blue staining[20] and silver staining[21]. Protein concentration was estimated by Bradford’s microassay using bovine serum albumin as standard [22]. HpDprA was purified as described previously [16].

**Table 1:** Sequences of the oligonucleotides used in this study.

oligonucleotide	Sequences 5ʹ-3ʹ
Oligo 1	GTC**GGATCC**ATGAAAAGCAATTTCCAATAC
Oligo 2	CTTT**CTCGAG**TCATTTTAAAAGGGTGTTTATAAAATTTTGAAT
Oligo 3	GTC**GGATCC**GATTACCATTTAAAAGAAATGCCT
Oligo 4	CTT**CTCGAG**TCATGCTAACACCACGAGATG
Oligo 5	CGAGATCGGGGGATCAGCAGTCTAGACCAGGTCAGCCGCGAGGACGACAGCAGTCTAGACCTTGGCGTAATCATGGTCAT
Oligo 6	ATGACCATGATTACGCCAAGGTCTAGACTGCTGTCGTCCTCGCGGCTGACCTGGTCTAGACTGCTGATCCCCCGATCTCG
Oligo 7	TCGCGCGTTTCGGTGATGACGGTGAAAACCTCTGACACATGCAGCTCCCGGAGACGGTCACAGCTTGTCTGTAAGCGGATGCCGGGAGCA biotin
Oligo 8	TGCTCCCGGCATCCGCTTACAGACAAGCTGTGACCGTCTCCGGGAGCTGCATGTGTCAGAGGTTTTCACCGTCATCACCGAAACGCGCGA
Oligo 9	GTGGCTATCATAGGCACAGCAGCACCCACCCCTTACAGCAAGC
Oligo 10	GCTTGCTGTAAGGGGTGGGTGCTGCTGTGCCTATGATAGCCAC
Oligo 11	GAAAAGGATTTCATGCCCATTGCCGGCTCTTTTTTAGCCAGAAAC
Oligo 12	GTTTCTGGCTAAAAAAGAGCCGGCAATGGGCATGAAATCCTTTTC

### MALDI-MS analysis

MALDI–MS analysis to confirm mass of the purified proteins was carried out as described earlier [23].

### Oligonucleotides and radiolabeling

All oligonucleotides used in this study were synthesized by Sigma, Genosys. The concentrations of oligonucleotides were determined by UV absorbance at 260 nm. Extinction coefficient of oligonucleotides was calculated using the sum of the extinction coefficients of the individual bases. The oligonucleotides were labeled at the 5’ with [ᵞ^32^P] ATP using T4 polynucleotide kinase and purified by Qiagene nucleotide removal kit. For experiments with ssDNA oligo 5 (80 mer) was labeled (Table 1) as described above. Duplex dsDNA was formed by first labelling oligo 5 and subsequently annealing the labelled oligo with excess of oligo 6. Annealing reactions were carried out in 1X saline sodium citrate buffer.

### Nuclease cleavage assays

Nuclease cleavage assays were performed in a 20 µl reaction mixture containing 0.5 nM DNA substrates (^32^P-ssDNAor ^32^P-dsDNA) in reaction buffer (recommended NEB Buffer for each respective nuclease) and indicated concentrations of HpDprA. The cleavage reaction was started with the addition of respective endonucleases (1U/reaction). Digestion was carried out for 30 min at 37 °C. Reaction was stopped with the addition of 10 mM EDTA and the samples deproteinized by the action of Proteinase K (10 μg/reaction) in the presence of 0.05% SDS for 15 min at 65°C. Degraded DNA was separated from protected DNA on a denaturing (7 M urea) 8% polyacrylamide gel (0.5X TBE). A constant voltage of 14 V/cm was applied for 3 h at room temperature. The gel was visualized by phosphorimaging analysis of the dried gel. (Fujifilm FLA-9000).

### Surface Plasmon Resonance studies

The binding kinetics of HpDprA and its variants with DNA was determined by SPR spectroscopy using the BIAcore3000 optical biosensor (GE Healthcare Lifescience) as described previously [24] with certain modifications explained here. A 3′-end-biotinylated 90-mer single stranded DNA (oligo 7; Table 1) and a 3′-end-biotinylated 90-bp double stranded DNA (formed by annealing oligo 7 with oligo 8; Table 1) was immobilized on the surface of a streptavidin-coated sensor chip (GE Healthcare Lifescience) to a final concentration of ~1000 response units (RU) / flow cell. The binding reactions were carried out at 25 °C in a continuous flow of standard buffer containing 10 mM HEPES buffer (pH 7.4) 150 mM NaCl, 1 mM EDTA, and 0.05% surfactant P-20 at a flow rate of 20 μl/min. Increasing concentrations of HpDprA and its variants were injected onto the surface of the biosensor chip for 100 s at a flow rate of 20 μl/min, followed by a dissociation period of 300 s. The required protein concentrations were made by diluting with standard buffer. The surface was regenerated by using the running buffer. One of the four surfaces without the biotinylated oligonucleotide was used as negative control. Background nonspecific binding and the bulk concentration were experimentally determined by simultaneous injections over a surface that lacked DNA. The association and dissociation of the protein from DNA were monitored by changes in resonance due to the change in mass on the sensor surface. Each experiment was repeated at least thrice to ensure reproducibility of results. Since the observed dissociation kinetics for DNA-protein interaction was biphasic, the standard Langmuir binding equation did not result in a good fit for the dissociation phase. However a decent fit was obtained for association kinetics using Langmuir model. Hence, the on-rate K_on_ (M^-1^s^-1^) was calculated using 1:1 langmuir binding model of BIAevaluation software (version 3.0). The off rate K_off_ (s^-1^) for each injected concentration was calculated according to the equation: *f* = *y*_0_ + *a* ^−*bx*^ + *c* ^−*dx*^ using Sigma plot (version 11.0). Two off rates were obtained for a biphasic dissociation curve. Out of these two off rates, the one belonging to the phase with maximum signal fall was considered for the calculation of dissociation constant K_d_. The dissociation constant K_d_ was calculated according to the equation:

$$k_{d}=k_{off}/k_{on}.$$

To study the homotopic protein interactions, HpDprA, HpRF and HpDML1 was immobilized on the surface of a CM5 sensor chip (GE Healthcare Lifescience) using 1-ethyl-3-(3- dimethylaminopropyl)-carbodiimide and N-hydroxysuccinimide activation chemistry, as per the manufacturer's instructions. After coupling of the protein, the unbound carbodiimide groups were blocked with ethanolamine. Coupling of individual proteins to the CM5 surface resulted in a response signal of ~1000 RU / flow cell. One of the flow cells was treated with ethanolamine alone to serve as reference channel for nonspecific binding. Binding experiments were carried out in standard buffer at a flow rate of 20 μl/min at 25 °C. Different concentrations of the proteins were injected over the flowcell surface, followed by a dissociation period of 300 s. The surface was regenerated by using the running bufer. All the data collected for the protein–protein interactions were corrected for nonspecific binding in the blank reference flow cell. The binding data were analyzed as explained for DNA–protein interaction.

### Circular dichroism spectroscopy

Far-UV Circular dichroism (CD) spectra were recorded using a Jasco-815 spectropolarimeter equipped with a Peltier stage (Japan Spectroscopic Co., Japan) at 25°C. The spectra were recorded from 200 to 300 nm with 20 mdeg sensitivity at a scan speed of 50 nm using 0.1 mg/ml protein in a 2 mm path length cuvette with a bandwidth of 2 nm. The experiments were carried out in 137 mM NaCl, 2.7 mM KCl, 10 mM Na_2_HPO_4,_ 1.8 mM KH_2_PO_4_ (1X PBS–pH 7.4). Each spectrum is an average of at least 3 scans. The spectra were corrected with the respective buffer control. The obtained ellipticity values were converted to Mean Residual Ellipticity [θ MRE], by using following equation: $[\theta MRE]=\frac{\theta \times 100\times M_{r}}{C\times L\times NA}$

Mean residual ellipticity (degrees)

M_r_ = molecular weight of the protein in Dalton

L = Path length in cm

NA = number of amino acids

The secondary structure of individual proteins have been calculated from respective CD spectra using K2D2, a web server to estimate the α helix and β strand content of a protein from its circular dichroism spectrum [25].

### Gel filtration analysis

Gel filtration experiments were performed using Superdex75 HR 10/300 column (GE healthcare) connected with AKTA basic 10 liquid chromatography system (GE healthcare). Chromatography was carried out in 1X PBS at a flow rate of 0.3 ml / min. The column was equilibrated using standard molecular weight markers (Bio-Rad, USA): Thyroglobulin (670 kDa), γ- globulin (158 kDa), ovalbumin (44 kDa), Myoglobuling (17 kDa), and vitamin B12 (1.35 kDa). The void volume (V_o_) was determined using Blue-dextran. Since HpDprA shows very low absorption at 280 nm, the elution volume (V_e_) for each protein was determined by monitoring absorption at 230 nm and 280 nm. The elution profile measured at 230 nm and 280 nm perfectly overlapped with each other (data not shown). A standard curve was obtained by plotting logarithmic molecular weight against V_e_/ V_o._

### Chemical cross linking

HpDprA and its individual purified domains were subjected to chemical–crosslinking by gluteraldehyde as described previously [26]. Indicated concentrations of each of the proteins were incubated on ice with different concentrations of gluteraldehyde (as shown in figure legends). The reactions were stopped by adding SDS sample buffer and were fractionated on 0.1% SDS– 10% PAGE for HpDprA and HpRF and 0.1% SDS– 12% PAGE for HpDML1. The protein bands were visualized by silver staining.

### Atomic force microscopy

Atomic force microscopy was done as described earlier [15,17] with some modifications as described. HpDprA or HpRF was incubated with 5 nM circular supercoiled pUC19 plasmid DNA in a buffer containing 20 mM Tris (pH 7.5), 50 mM NaCl, 5 nM MgCl_2_ and 50 µM spermidine. 10 µl of the sample was deposited on a freshly cleaved mica surface and incubated for 90 seconds. The surface was rinsed with ultrapure water and dried. Images were acquired in air with NX—10 AFM (Park systems) operated in non- contact mode. Though most of the AFM imaging with biological samples is carried out in tapping mode, Park system AFM offers only two modes of imaging i.e., contact and non–contact mode. A stiff cantilever used in non-contact mode of the XE-series park system AFM detects changes in the resonant frequency or vibration amplitude as the tip nears the surface conferring it sub-angstrom vertical resolution of the image, as with contact mode (http://www.parkafm.com/images/spmmodes/standard/True-Non-Contact-Mode.pdf). Hence, specifically for this system non-contact mode is suitable for achieving high resolution image of a soft biological sample. A silicon nitride cantilever was used with a force constant of 42 N/m. Imaging was done at a resolution of 512 X 512 pixels. Raw data was selected with the XEI image processing programme and the same was used to flatten AFM images with first order polynomial fitting. The height and width of DNA alone and complex was calculated using XEI image processing programme. Each experiment was repeated at least three times and standard deviation was calculated.

### Construction and purification of DprA mutants using site directed mutagenesis

The mutants of DprA were constructed using QuickChange II site directed mutagenesis kit (Agilent technologies). Primers and PCR reaction were designed according to the manufacturer’s recommendation. Double mutant (HpDprA^R48A/R49A^) was constructed using Oligo 9 as forward primer and Oligo 10 as reverse primer (Table 1). Double mutant was used as a template to construct triple mutant (HpDprA^R48A/R49A/K133A^) using Oligos 11 and 12 (Table 1). Mutation of basic amino acids to non-polar amino acids results in change of pI from 8.55 to 7.64 for double mutant and to 6.91 for triple mutant. Hence, to ensure the solubility of mutants during the purification the pH of buffer was kept 6.5. Purification of both mutant proteins was carried out using Ni-NTA and Heparin column as described for wild type DprA [16].

## Results

### HpRF is sufficient for binding ssDNA or dsDNA albeit with lower affinity

To understand the role of individual domains of HpDprA, both domains were purified separately. A polypeptide containing 1–212 amino acids was expressed as a soluble recombinant protein with an N-terminal (His)_6_ tag in *E*. *coli* and purified to near homogeneity (Fig A part A in S1 File). Similarly, a polypeptide containing 213–266 amino acids was purified (Fig A part B in S1 File). The theoretical mass of HpDprA is 33.36 kDa, HpRF is 26.90 kDa and HpDML1 is 10.20 kDa. The masses of both purified domains were confirmed by MALDI-MS analysis (Fig A part C and D in S1 File). HpRF comprises the Rossmann fold domain from the full length protein and HpDML1 contains most of the linker region (7 out of 8 amino acids) and the DML-1 like domain. Circular dichroism spectroscopy was used as a probe to determine secondary structure content of HpDprA, HpRF and HpDML1. Far-UV CD spectrum of HpDprA collected at 25˚C displayed an appreciable contribution of α-helical structure with two minima observed at 222 nm and 209 nm (Fig A part E in S1 File). The tertiary structure of HpDprA has been shown to be rich in α –helical content [10]. The obtained CD spectrum matches with earlier published data (Table 2). The observation of similar secondary structure composition for the HpDprA and HpRF in the HpDprA structure (Table 2) was confirmed by Far UV- CD spectrum of the two proteins which show a good overlap (Fig A part E in S1 File). Therefore, the purified HpRF did not have any gross structural alteration. The Far-UV CD spectrum of HpDML1 depicted a higher contribution of β sheets than α –helix (Fig A part E in S1 File, Table 2). Earlier published data shows presence of higher α –helical content in HpDML1 (Table 2). However, crystal structure data is not available for HpDML1 and moreover, Wang et al [10] predicted the structure of HpDML1 through I–TASSER.

**Table 2:** Secondary structure composition of full length HpDprA and its individual domains.

Secondary structure	HpDprA	HpRF	HpDML1	
% α - HELIX	37%	37%	37%	From crystal structure
	50.16% (0.4)	52% (0.4)	6.71% (0.38)	From CD spectra
% β - SHEET	15%	15.5%	13%	From crystal structure
	7.41% (0.4)	8.21% (0.4)	32.75% (0.38)	From CD spectra

The secondary structure of individual proteins have been calculated using K2D2, a web server to estimate the α helix and β strand content of a protein from its circular dichroism spectrum and compared with the % α –helix and % β sheet calculated from HpDprA structure published by Wang et. al., (2014). RMSD values depicting goodness of fit has been shown in bracket**.**

**Fig 1:**
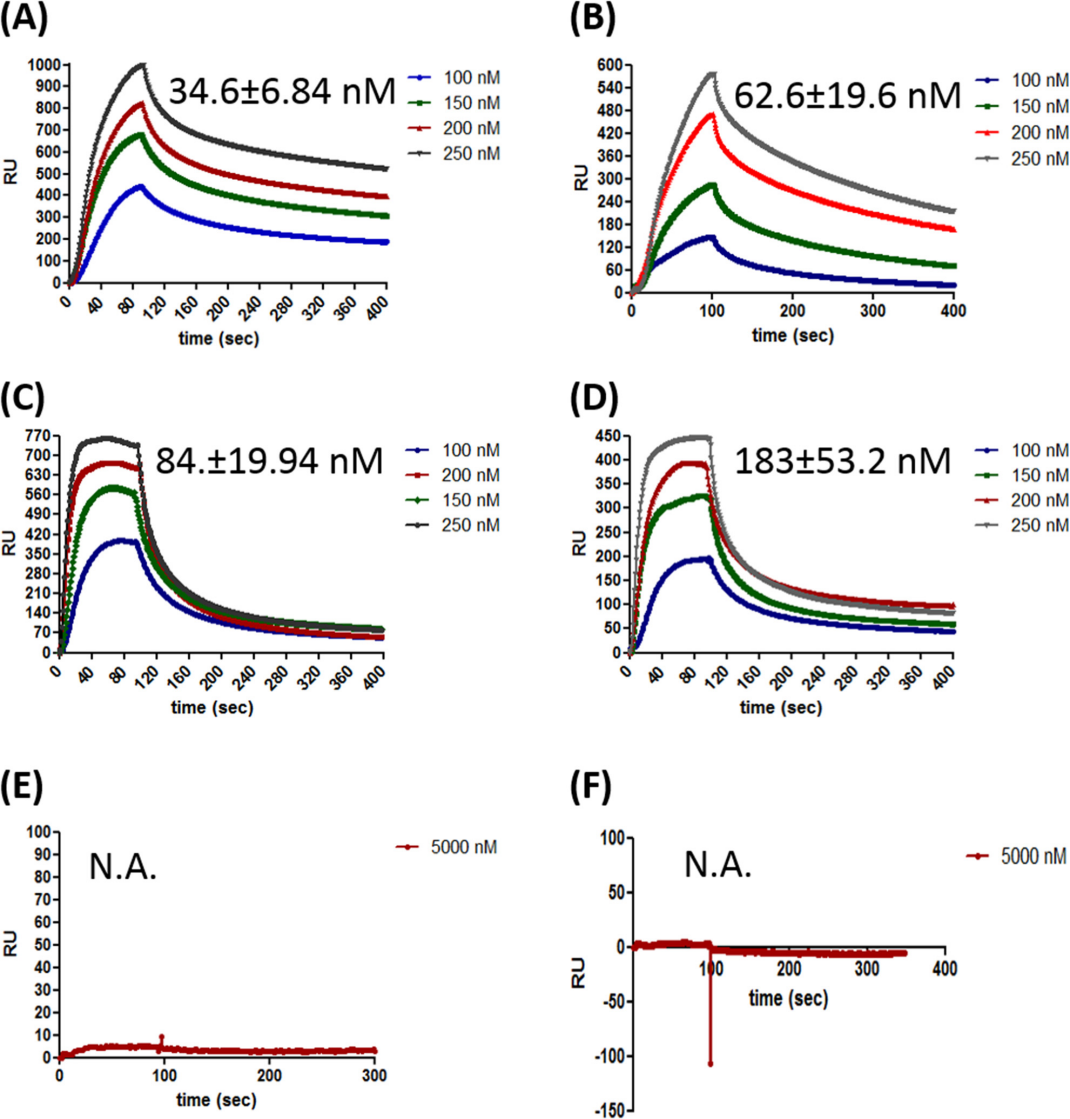
Surface plasmon resonance analysis of HpDprA, HpRF and HpDML1 interaction with DNA. Different concentrations of full length HpDprA **(A)**HpRF**(C)** and HpDML1**(E)** were injected on to the immobilized single stranded DNA (90 mer, ~1000 RU) surface in buffer containing 150 mM NaCl. Both sample injection and buffer injection were carried out as described in materials and methods. Similarly, binding sensorgrams were obtained for the interaction of dsDNA (90 bp, ~1000 RU) with full length HpDprA **(B)**HpRF**(D)** and HpDML1**(F)**. Sensorgrams depicting changes in response unit (the y—axis) as a function of time (the x—axis) are shown. The concentrations of soluble analytes and affinity constant (K_d_) values are indicated in the inset to the figures.

HpRF was shown to be sufficient for binding ssDNA [10]. However, the full length protein was shown to have higher affinity for ssDNA than HpRF[10]. Two crucial questions are addressed here using Surface plasmon resonance. (a) Is HpRF sufficient to bind dsDNA as well? (b) What is the difference in binding kinetics of full length protein as compared to HpRF with respect to ssDNA and dsDNA? ssDNA and dsDNA were immobilized on SA sensor chip (streptavidin coated sensor chip) separately and varying concentrations of HpDprA, HpRF and HpDML1 were passed through a BIAcore flow cell. HpDprA displayed high affinity (K_d_ = 34.6 nM) for ssDNA (Fig 1A). HpDprA showed nearly two fold lower affinity for dsDNA (K_d_ = 62.6 nM) as compared to ssDNA (Fig 1B). An interesting observation was made from the binding sensogram of HpRF with ss and ds DNA. HpRF showed faster association kinetics for interaction with ssDNA (Fig 1C) and dsDNA (Fig 1D). The binding saturation was observed within ~20 sec fromthe start of the injection for ssDNA. However HpRF complex with ssDNA or dsDNA was not stable and much faster dissociation of complex was observed in comparison with full length protein (compare dissociation phases of Fig 1A with 1C and 1B with 1D). The dissociation constant of HpRF was ~ 2.5 fold higher as compared to full length protein for ssDNA (K_d_ = 84.1 nM) and ~ 3 fold higher for dsDNA (183 nM). Since the association rate (K_on_) for HpRF–DNA interaction is higher, the dissociation constant (K_off_/K_on_) values obtained are lower. The slower association rate of HpDprA could be due to steric hindrance offered by HpDML1. The actual affinity of HpRF for DNA may be even lower.

The higher stability of full length HpDprA complex with ss and ds DNA could be reasoned through two different hypothesis (a) HpDML1 possesses a secondary DNA binding site. Therefore full length DprA–DNA interaction is characterized by two binding events and thus takes longer time to reach binding equilibrium (b) interaction of full length HpDprA with DNA may be intricate involving different kind of interactions (protein–protein and DNA—protein) resulting in a higher order complex formation. Such kind of complex would take longer time to form as compared a DNA-protein complex formation involving just electrostatic interaction. SPR was carried out to check binding of HpDML1 with ssDNA and dsDNA. The binding sensorgrams showed lack of binding of HpDML1 with ssDNA (Fig 1E) or with dsDNA (Fig 1F). Thus, SPR experiments ascertained that there is no secondary binding site present in HpDML1 for ssDNA or dsDNA interaction. The inability of HpDML1 to bind DNA (either ss or ds), eliminates the first possibility. Hence, the observed differences in DNA binding sensorgrams between full length HpDprA and HpRF could be because of the mechanism discussed in (b), proposing the interaction of HpDprA with DNA involving different kind of associations, DNA–protein and protein–protein, resulting in higher order complex formation.

### HpDprA can oligomerize using two different sites of interaction

**Fig 2:**
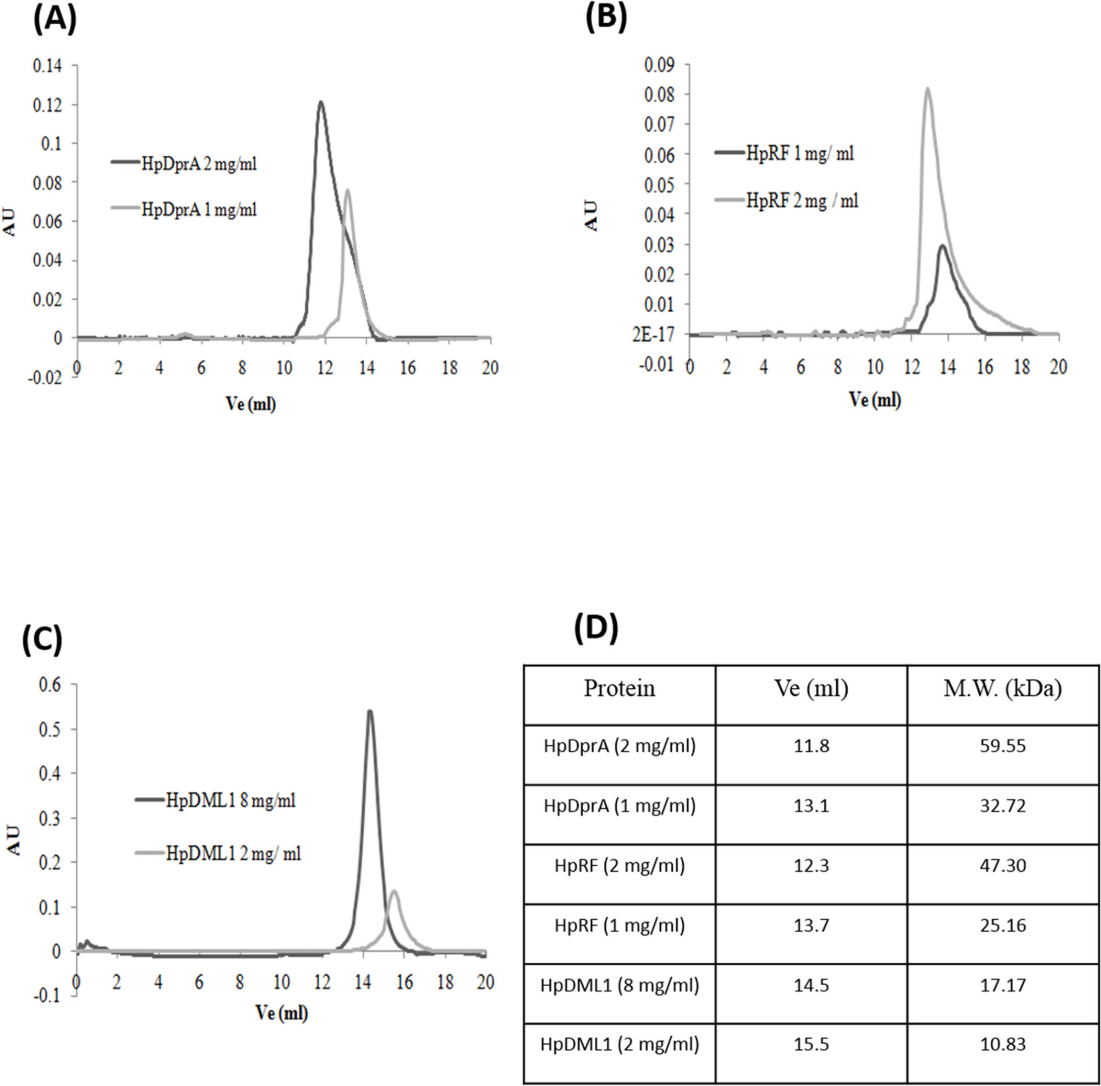
Determination of oligomeric status of HpDprA and its domains. Elution chromatograms of purified HpDprA at 2 mg/ml and 1 mg/ml **(A)** concentrations are depicted. Similarly, the size exclusion profiles of HpRF at 2 mg/ml at 1 mg/ml **(B)** and HpDML1 at 8 mg/ml at 2 mg/ml **(C)** are depicted. **(D)** Table shows elution volume (V_e_) and corresponding molecular weight of the elution peaks shown in (**A, B** and **C**). Elution was monitored using UV absorbance at 230 nm wavelength. The column was equilibrated in the same buffer as the protein samples (1X PBS). Total 500 µl sample was injected for each chromatophic experiment. The apparent molecular mass was calculated from elution volume using molecular weight standards as described in materials and methods.

It has been shown for SpDprA [13] and HpDprA [10] that dimerization is crucial for their interaction with ssDNA. The dimerization motif was mapped to the HpRF domain. To characterize the oligomerization property of HpDprA, different concentrations of protein were passed through superdex—75 gel filtration column calibrated using proteins of known molecular masses. HpDprA was found to elute with an apparent mass of 59.55 kDa at 2 mg/ml concentration (Fig 2A and 2D). However, at lower concentration (1 mg/ml), HpDprA was found to elute faster with peak corresponding to the molecular mass of 32.72 kDa (Fig 2A and 2D). Similar observations were made for HpRF where the median of peak shifts more towards the dimeric population at 2 mg / ml concentration as compared to 1 mg / ml concentration (Fig 2B and 2D). The data obtained from size exclusion chromatography indicates that for both HpDprA and HpRF, dimeric populations are more favoured at higher concentrations. Thus, HpDprA dimerization may rely primarily on N-N interaction (HpRF-HpRF interaction). HpDML1 of HpDprA eluted from the sizing column with an apparent mass of a monomer (10.83 kDa) at 2 mg/ml concentration (Fig 2C and 2D). Further injection of HpDML1 at four fold higher concentrations (8 mg/ml) resulted in a dimeric peak (volume corresponding to mass 17.17 kDa) (Fig 2C and 2D). SpDprA forms filament like packing during crystallization in which both Rossmann fold domain and Sterile Alpha Motif domain self-interactions were observed [10]. Hence, it is possible that HpDprA may form higher oligomeric forms using more than one interface.

To further substantiate the observations made from size exclusion chromatography, chemical cross linking of HpDprA, HpRF and HpDML1 using gluteraldehyde was performed. HpDprA (5 µM) was incubated with increasing concentrations of gluteraldehyde (0.01% to 0.5%) at 4˚C for 15 mins. At 0.01% gluteraldehyde concentration dimeric (66 kDa) and trimeric (99 kDa) species of HpDprA were observed (Fig B part A in S1 File, lane 2). At higher concentration of gluteraldehyde (0.05% and 0.1%) polymeric forms of HpDprA sitting in the well were observed along with dimeric and trimeric species (Fig B part A in S1 File, lane 3 and 4). At 0.5% gluteraldehyde concentration only dimeric and polymeric form of HpDprA was observed (Fig B part A in S1 File, lane 5). Therefore, the observed trimeric form is an intermediate between dimeric and polymeric species of HpDprA. Interestingly, in presence of gluteraldehyde only dimeric and no higher oligomeric form was observed for HpRF (Fig B part B in S1 File). This result suggests that presence of HpDML1 is needed for the formation of higher oligomeric forms of HpDprA. HpDML1 formed a dimer at a much higher protein concentration (140 µM) and longer incubation with cross linker was required (Fig B part C in S1 File). Similar to HpRF, HpDML1 also did not show any higher oligomeric form beyond a dimer. Gluteraldehyde cross linking results showed that while full-length HpDprA can form higher oligomeric forms (trimeric to polymeric), both HpRF and HpDML1 can only form dimer (the later one forms dimer with much lower efficiency than the former).

**Fig 3:**
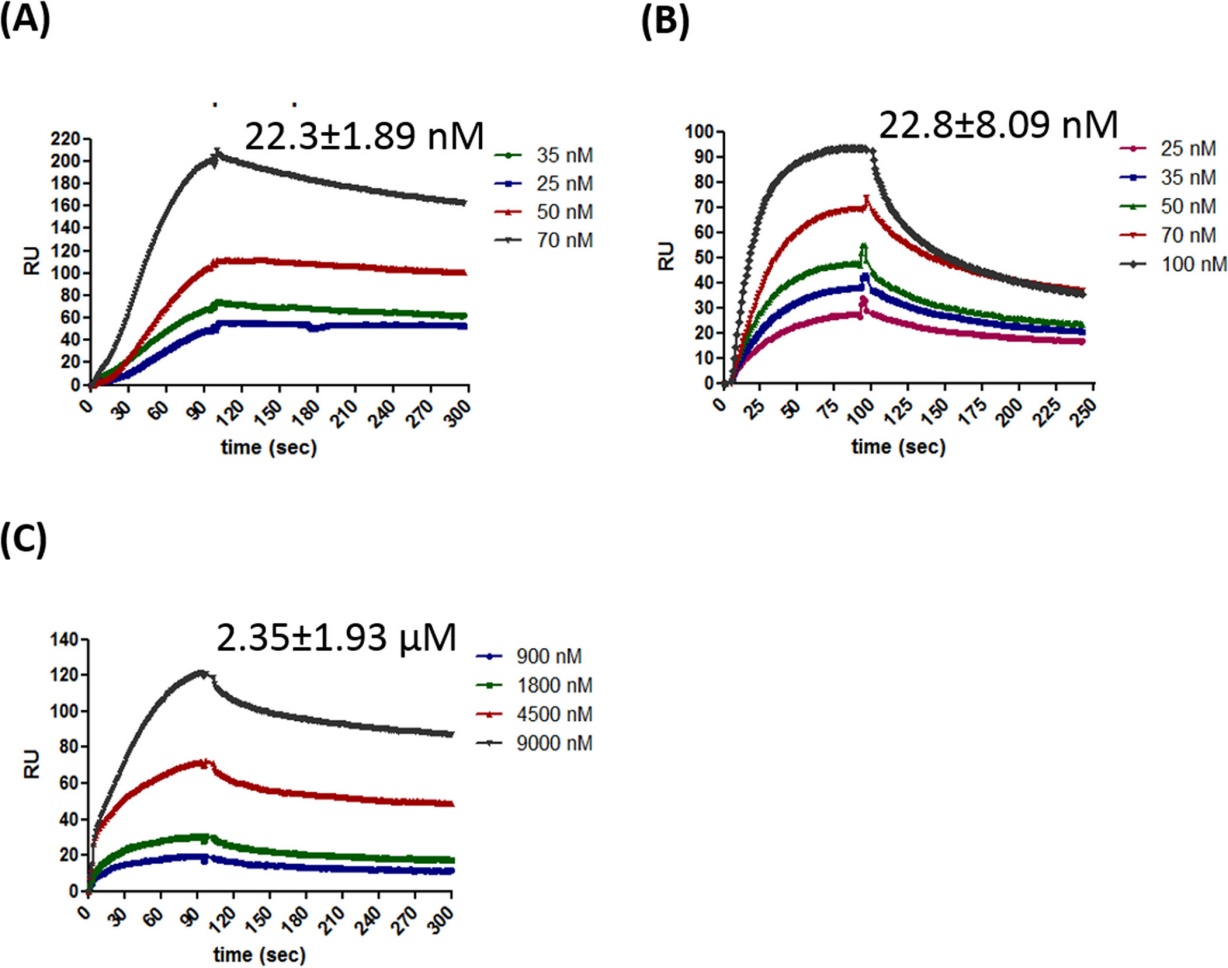
Surface plasmon resonance analysis of homotopic protein interaction between full length HpDprA, HpRF and HpDML1. Sensorgrams showing self-interaction of **(A)** HpDprA **(B)**HpRF and **(C)**HpDML1. The concentration of soluble analytes and affinity constant (K_d_) values are indicated in the inset to figures. The K_d_ values are determined as described in materials and methods.

To characterize the binding affinities of individual domains and full length protein SPR was carried out. HpDprA, HpRF and HpDML1 were immobilized on CM5 sensor chips and varying concentrations of the full length protein and its individual domains were passed through BIAcore flow cell. HpDprA showed very high affinity (K_d_ = 22.3 nM) for homotopic interaction (HpDprA—HpDprA) (Fig 3A). HpDprA formed a very stable complex resulting in very slow (but measurable) off rate. HpRF showed faster association profile reaching binding equilibrium faster than full length protein. The dissociation rate of HpRF was also high resulting in similar value of dissociation constant (K_d_ = 22.8 nM) as that of full length protein (Fig 3B). Measurable interactions for HpDML1 were obtained at much higher concentrations of protein resulting in a much higher dissociation constant (Fig 3C). HpDML1 displayed very low affinity for homotopic interaction as measured by binding sensorgrams of SPR. However, no interaction was observed between HpRF and HpDML1 (data not shown). Taken together, gel filtration, glutaraldehyde cross linking and SPR analysis suggest that HpDprA has two interfaces for oligomerization: (a) a higher affinity N-N interaction site (22.8 nM) and (b) a lower affinity C-C interaction (2350 nM) site.

### Binding of HpRF to ssDNA and dsDNA results in protection of DNA from nucleases

**Fig 4:**
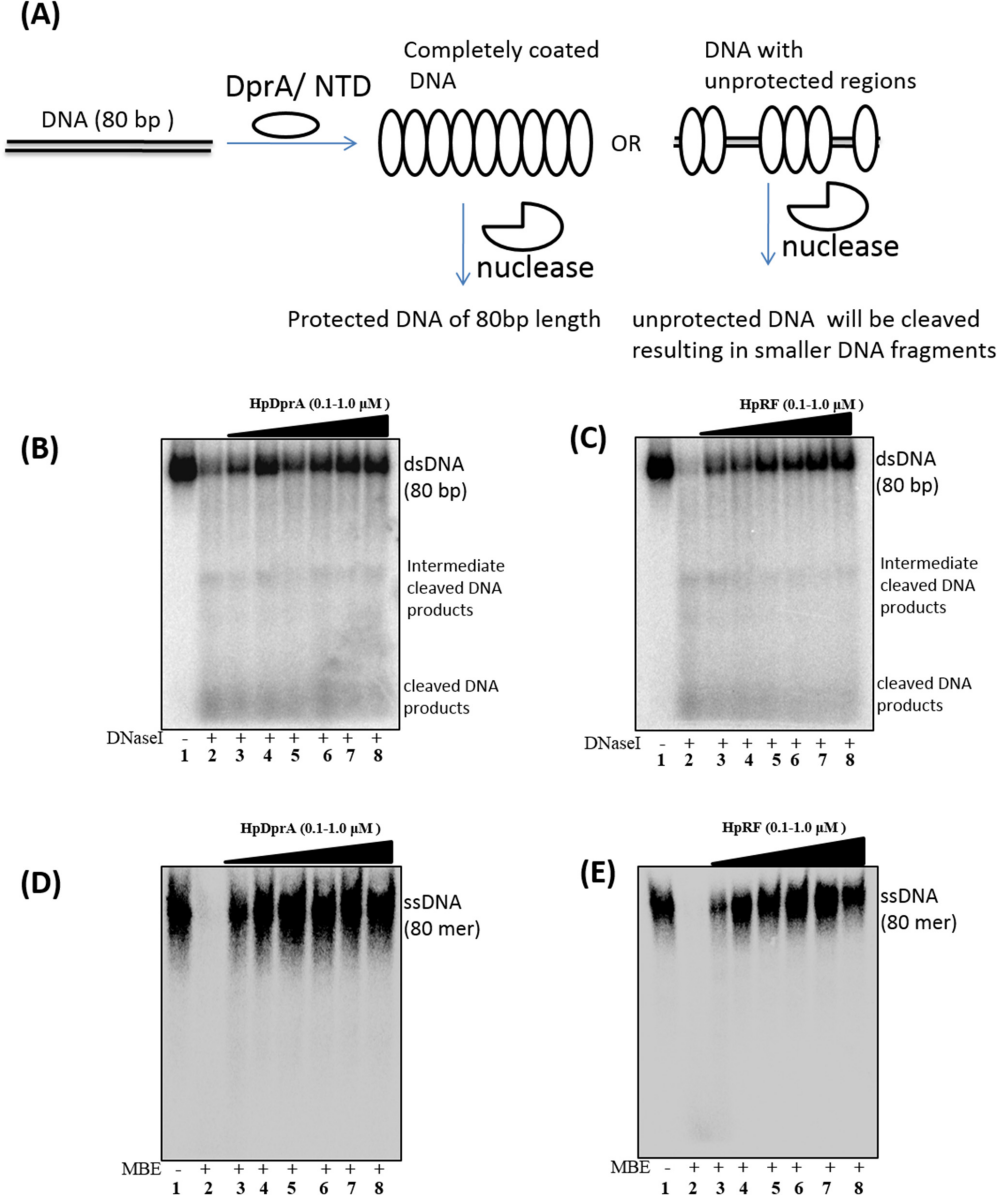
Nuclease protection assay. **(A)** Schematic illustration of nuclease protection assay. ^32^P-labeled dsDNA (0.5 nM) either alone (lane 2) or pre-bound with increasing concentrations of HpDprA **(B)** or HpRF**(C)** {100, 200, 400, 600, 800, 1000 (nM), lanes 3–8} was incubated for 30 min with 1 unit of DNaseI. Similarly, ^32^P-labeled ssDNA (0.5 nM) either alone (lane 2) or pre-bound with above mentioned concentrations of HpDprA **(D)** or HpRF**(E)**was incubated for 30 min with 1 unit of mung bean endonuclease. Lane 1: DNAalone.

HpDprA offers protection to bound ssDNA and dsDNA from various nucleases [16]. In the current study, role of HpRF in protection of bound ssDNA and dsDNA was investigated. As shown in Fig 4A, dsDNA was pre-incubated with HpDprA or HpRF and the complex was then treated with DNaseI (dsDNA specific nuclease). Similarly ssDNA was pre-incubated with HpDprA or HpRF and the complex was then treated with Mung bean endonuclease (ssDNA specific nuclease). As can be seen in Fig 4B and 4C, HpRF protects the dsDNA from DNaseI as effectively as full length protein. Similar observations were made for protection of ssDNA from mung bean endonuclease (compare Fig 4D with 4E). Nuclease protection assays demonstrate that HpDML1 is not necessary for protection of bound DNA. For protection from nucleases complete coating of bound DNA by protein is necessary. Hence, HpRF might be sufficient for forming nucleo–protein filament with ssDNA and dsDNA. It is known that positively charged surface of HpRF is involved in interaction with ssDNA [10], the other surface of protein might be involved in formation of nucleo- protein filaments. If role of HpDML1 is considered limited to a single nucleoprotien filament, then there are two interactions involved for formation of single filament i.e., DNA–protein and protein–protein interaction. Deletion of HpDML1 would result in a weaker nucleoprotein filament as the protein—protein interaction is now missing. Hence, the nuclease would get higher access to DNA when HpDML1 is deleted. But if HpDML1 plays role in cross interaction with two different nucleoprotein filaments, then deletion of it should not affect the strength of single filament. Hence, when challenged with nucleases, one would not be able to discriminate between these two different kinds of complexes formed by either HpDprA or HpRF. As shown in Fig 4, HpRF is equally efficient in protection of ss or dsDNA from nucleases as that of HpDprA suggesting a role of HpDML1 in inter-nucleoprotein filament associations.

### Deletion of HpDML1 results in reduction of inter-nucleoprotein filament interaction

**Fig 5:**
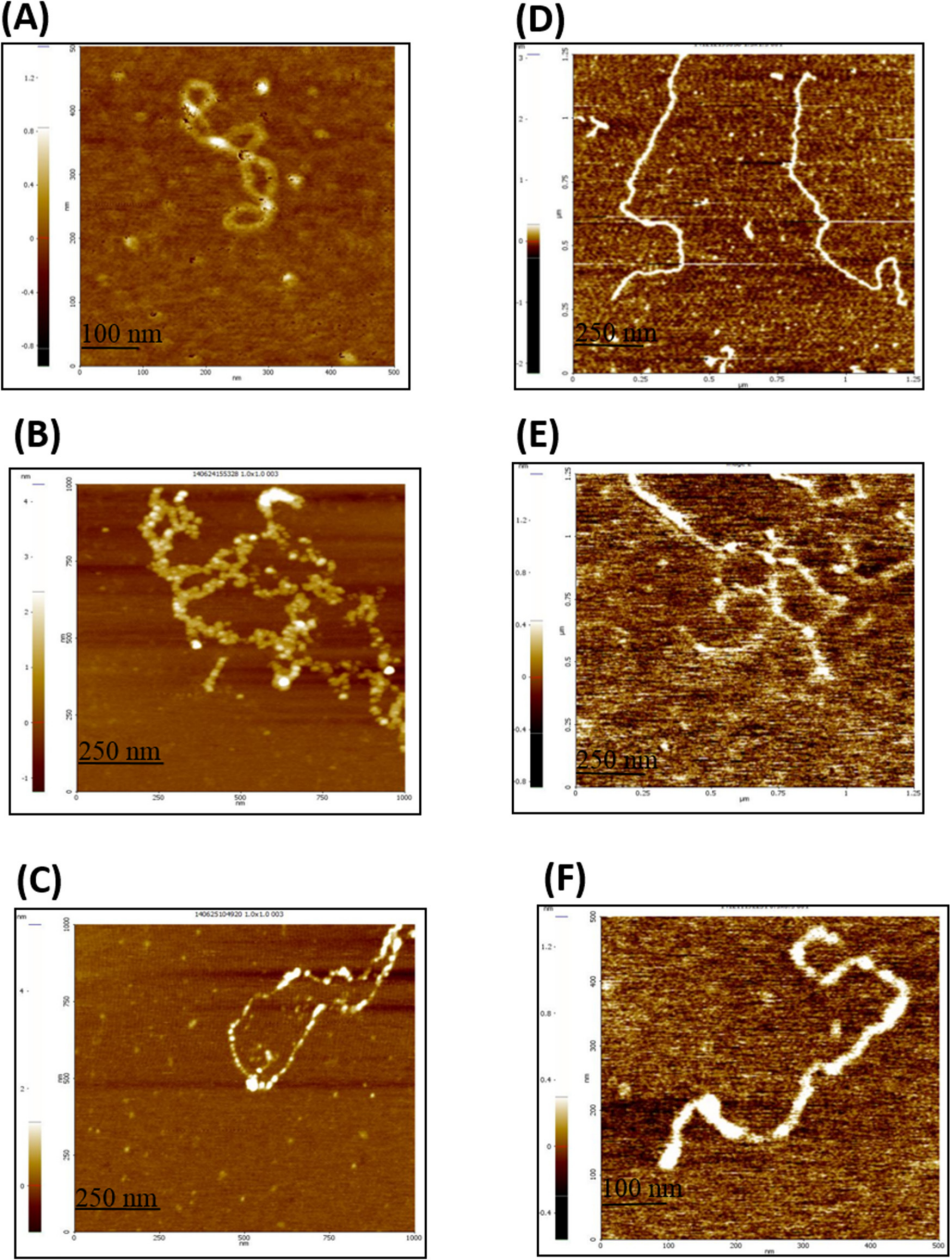
Atomic force microscopy illustrates that DprA forms a highly condensed complex but HpRF forms a less condensed complex with DNA. Reaction mixture contained 5 nM of circular supercoiled pUC19 DNA and 1 nM DprA or HpRF. Ten microliter aliquots were spotted on fresh mica and visualized as described in materials and methods. **(A)** Image of supercoiled pUC19 DNA. Image frame containing 1 nM HpDprA **(B)** or 1 nM HpRF**(C)** complex with pUC19 DNA **(D)** Supercoiled pUC19DNA has been linearized with SmaI restriction endonuclease. Complex of linear pUC19DNA with **(E)** 1 nM HpDprA and **(F)** 1 nM HpRF.

Nuclease protection assays shown in Fig 4, indicate a role for HpDML1 in inter- nucleoprotein filament interaction. To visualize such poly-nucleoprotein complex, AFM was utilized to probe HpDprA–DNA and HpRF–DNA interactions. For these studies, protein was incubated with supercoiled pUC19 DNA under optimal binding conditions. The AFM images of complexes were acquired in air. Fig 5A shows image of a circular supercoiled pUC19 DNA alone. The mean height and width of the pUC19 DNA calculated from the AFM scans were 0.5 ± 0.11 and 30 ± 3.5 nm respectively. An earlier reported height value of DNA correlates with this data [17]. However the measured width of any molecule in AFM experiments is influenced by curvature of radius of cantilever tip. In presence of HpDprA, highly condensed and interlinked large nucleoprotein complexes were observed (Fig 5B). The height of HpDprA–DNA complex measured was 2.075 ± 0.37 nm which is nearly four times higher than naked DNA. The average width of a single branch of complex was ~ 383.5 ± 38.37 nm nearly 10 times higher than naked DNA. The reported values of height and width here are comparable for most of the branches of complex observed, although many branches were thicker or thinner. The height and width measurements indicate a complex containing numerous molecules of protein and DNA packed together.

Homologous DprA proteins from other bacterial species were found to form dense complexes with ssDNA [15,17]. Transmission electron microscopy for SpDprA- ɸXssDNA complexes showed tightly packed discrete complexes that include numerous proteins [15]. AFM for BsDprA–ssDNA complexes revealed highly condensed ‘blob’ like structures [17]. Highly condensed complexes observed such as in Fig 5B could result by initial complete coating of DNA by DprA followed by many such nucleofilaments interacting extensively with each other resulting in highly compact, interlinked complexes. However, under the similar conditions HpRF alone formed very sparse complex with pUC19 DNA (Fig 5C) and could not induce condensation of DNA like full length protein. HpRF completely coated the DNA strand but unlike DprA- DNA complex extensive crosslinking between the nucleoprotein filaments was missing. The average width of pUC19 –HpRF complex was 50 ± 5.26 nm. Similarly, the average height of the complex was 0.8 ± 0.148 nm. The height and width measurements of HpRF–pUC19 DNA complex showed much thinner complex than that formed with HpDprA. The length of complexes (for both HpDprA and HpRF–pUC19 DNA) varied from one AFM snapshot to other depending upon number of individual filaments interacting with each other. However, for most of the images the length of HpRF- pUC19 DNA complex was more than that of the complex with HpDprA owing to lesser condensation in first case. SpDprA was observed to bind single stranded regions of supercoiled plasmid DNA [15]. To ascertain that the observed HpDprA binding with supercoiled plasmid DNA is not to its single stranded region and represents true dsDNA binding activity, the plasmid DNA was linearized using SmaIrestriction endonuclease (Fig 5D). Both HpRF and HpDprA were found to bind linear dsDNA by completely coating it (Fig 5E and 5F). In addition, similar to supercoiled dsDNA, HpDprA was found to form poly nucleoprotein complex involving many DprA–DNA complex with linear dsDNA as well (Fig 5E). However, HpRF formed a single discreet nucleo–protein filament with linear dsDNA (Fig 5F). AFM images clearly reveal that while HpRF can bind DNA by completely coating it, HpDML1 is required for inter-nucleoprotein filament interactions.

### Positively charged DNA binding pocket of HpDprA plays an important role in interaction of protein with ssDNA and dsDNA

**Fig 6:**
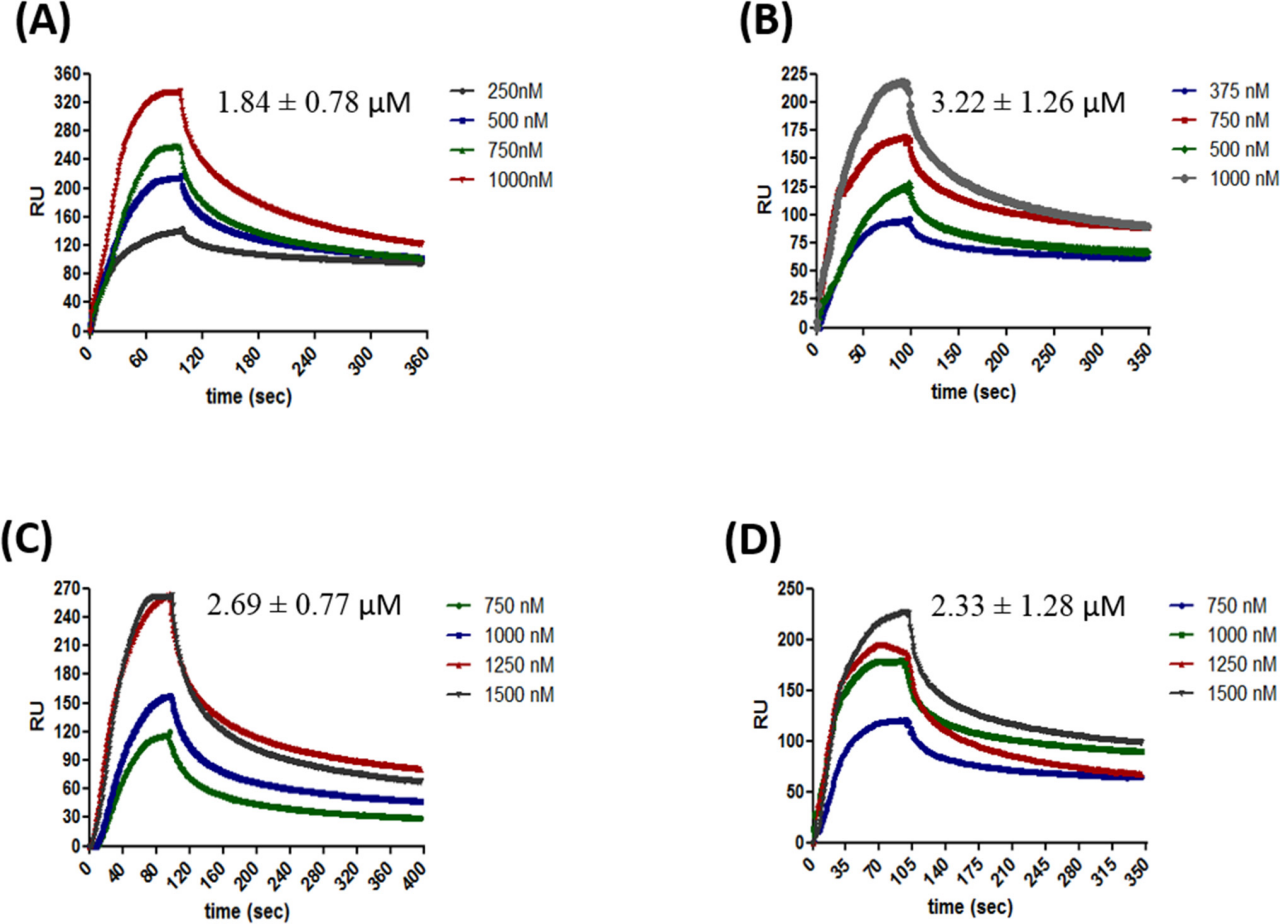
Surface plasmon resonance analysis of HpDprA mutants interaction with DNA. Different concentrations of **(A)**HpDprA^R48A/R49A^and **(C)**HpDprA^R48A/R49A/K133A^ were injected on the single stranded DNA surface in standard buffer containing 150 mM NaCl. Both sample injection and buffer injection were carried out as described in materials and methods. Representative sensorgrams illustrating changes in the response units (the y—axis) as a function of time (the x-axis) are shown. Similarly, binding sensorgrams were obtained for the interaction of dsDNA with **(B)**HpDprA^R48A/R49A^and **(D)**HpDprA^R48A/R49A/K133A^. The concentrations of soluble analytes and affinity constant (K_d_) values are indicated in the inset to the figures. The K_d_ values are determined as described in materials and methods.

Surface potential calculation showed that there were no obvious grooves on the HpDprA dimer, but a highly conserved positively charged area was shown to be the DNA binding site[10]. Mutation experiments indicated crucial role of basic amino acids Arg 52, Lys 137 and Arg 143 (Arg 48, Lys 133 and Arg 139 for HpDprA from strain J99) in HpDprA interaction with ssDNA[10]. However, the residues important for the interaction with dsDNA are not known. It is possible that the positively charged pocket of HpRF could be responsible for binding dsDNA as well or there could altogether be a different motif responsible for interaction with dsDNA. To investigate the role of positively charged surface of HpRF in dsDNA binding, a double mutant (HpDprA^R48A/R49A^) and a triple mutant (HpDprA^R48A/R49A/K133A^) were constructed and mutant proteins purified to near homogeneity (Fig C part A in S1 File). Far-UV CD spectrum of the double and triple mutant proteins overlapped well with that of wtHpDprA confirming that there were no major structural disturbances (Fig C part B in S1 File). The double mutant (HpDprA^R48A/R49A^) showed ~50 fold reduction in affinity for ssDNA binding (Fig 6A). Interestingly, the double mutant also showed ~35 fold reduction in binding affinity for dsDNA (Fig 6B). This illustrates that the residues important for interaction with ssDNA were also possibly involved in binding dsDNA. Further, mutation of lysine residue at 133 position (HpDprA^R48A/R49A/K133A^) resulted in nearly 75 fold reduction in ssDNA binding affinity (Fig 6C) and ~50 fold reduction in binding affinity for dsDNA (Fig 6D). The results obtained in our study demonstrate that these amino acids play an important role for binding single stranded and double stranded DNA. Nevertheless, the above mentioned three amino acids are more important for binding ssDNA than dsDNA, as it is evident from the fact that the mutations result in higher fold reduction in the ability of protein to bind single stranded DNA.

## Discussion

HpDprA consists of an N-terminal Rossmann fold domain and a C-terminal DML-1 like domain [10]. Both these domains are found to be prominently α-helical in nature. Amino acid sequence analysis of the protein suggests that HpRF is basic and HpDML1 is acidic in nature. HpRF is sufficient for binding with ssDNA and dsDNA, while HpDML1 plays an important role in formation of higher order polymeric complex with DNA. For HpDprA and SpDprA, dimerization interface was mapped to the Rossmann fold domain [10,13]. Gel filtration data revealed an important observation that HpDprA, HpRF and HpDML1 can exist as a dimer (dominant species at higher concentration) in solution. Interestingly, both domains of HpDprA i.e., HpRF and HpDML1 were able to form dimers but not higher oligomeric forms. On the other hand, HpDprA was seen to form oligomeric forms higher than dimer in gluteraldehyde cross linking assay. The strength of HpDML1 dimer was much lower than HpRF dimer, and hence we propose that there are two sites of interactions present in HpDprA—a primary interaction interface(N-N interaction) and a secondary interaction interface (C-C interaction). The N-N interface is responsible for dimer formation but further oligomerization of HpDprA necessitates the interaction of two dimers using C-C interaction interface.

**Fig 7:**
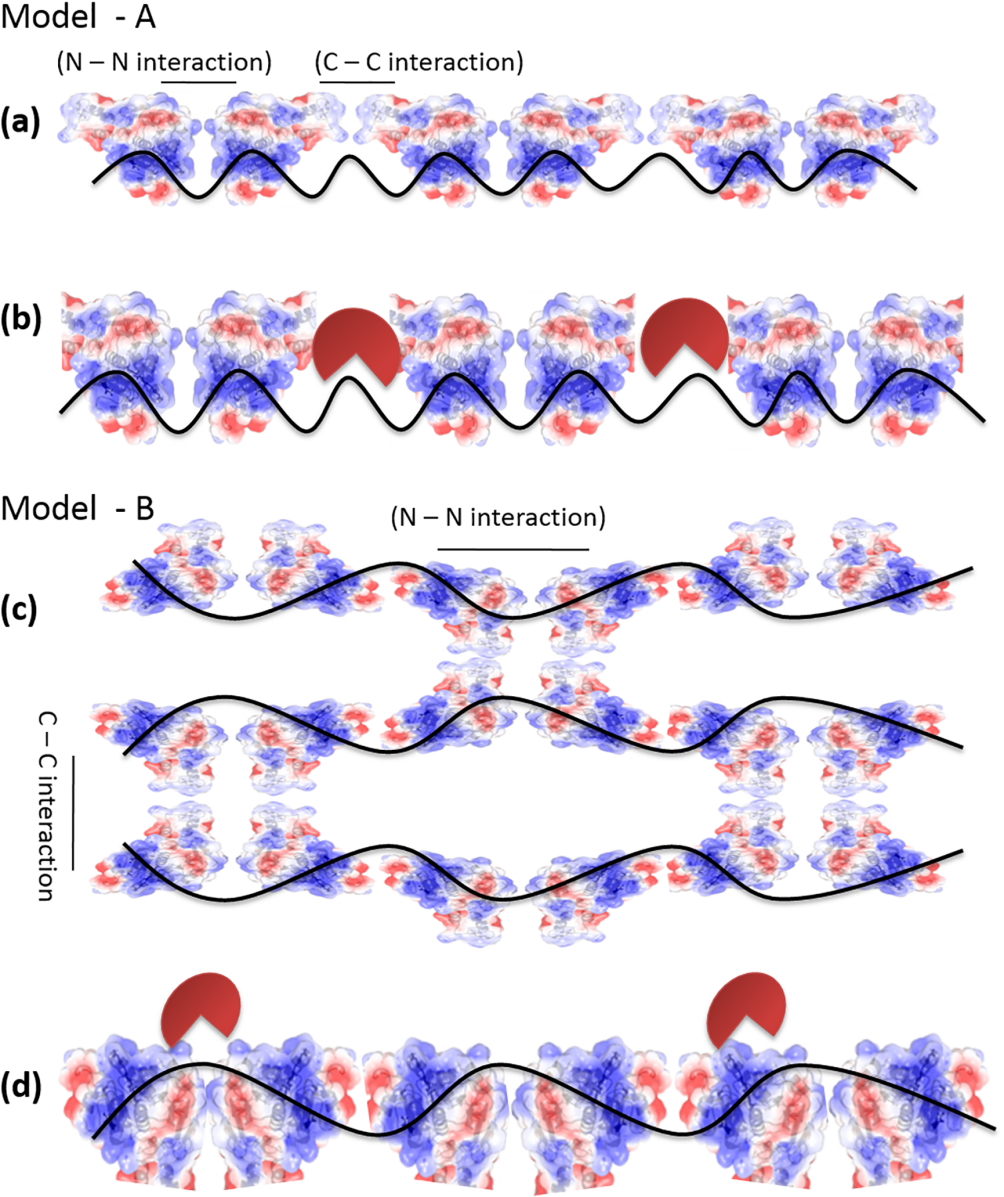
Possible roles of HpDML1 in nucleoprotein complex formed by interaction of HpDprA with DNA. **Model A:** C-C interaction bridges two dimers of HpDprA on DNA and thus allows the protein to oligomerize on DNA. **(a)**schematic describing the role of C-C interaction restricted to single nucleoprotein filament. **(b)** outcome of deletion of HpDML1 in nuclease protection assay if model A best represents the role of C-C interactions. **Model B:** C-C interactions play a role in inter–nucleoprotein filament interaction **(c)** schematic describing role of C-C interactions in cross interaction of two nucleo–protein filaments. **(d)**outcome of deletion of HpDML1 in nuclease protection assay if model B best represents the role of C-C interaction. The red colored pie shape indicates ss or dsDNA nuclease. The solid structure represent HpDprA and the black curved line represents ss or dsDNA.

It was shown that HpRF binds to ssDNA but forms a lower molecular weight complex [10]. SPR analysis of DprA and HpRF–DNA interaction pointed out that deletion of HpDML1 leads to faster dissociation of the protein from DNA. Concomitantly, reduction in binding affinity was observed for both ss and ds DNA upon deletion of HpDML1from full length protein (Fig 1C and 1D). These results suggest that HpDML1 does play an important role in interaction of full length HpDprA with DNA. Two possible roles of HpDML1 were proposed by Wang *et al* (2014) to explain their observation of formation of lower molecular weight complex in absence of HpDML1. (i) HpDML1 possesses a second DNA binding site but much weaker than site present in HpRF. (ii) HpDML1 is not involved in DNA binding but mediates nucleoprotein complex formation through protein–protein interaction [10]. SPR analysis with purified HpDML1 protein confirmed that there is no secondary DNA binding site present in HpDML1 (Fig 1E and 1F). As discussed before, it was observed that HpDML1 can mediate interaction between two HpDprA through C-C interaction. Since this interaction is weaker it is less likely to be responsible for dimer formation. However, it could play an important role in trimer or higher oligomeric form of HpDprA where presence of N-N interaction will facilitate and stabilize C-C interaction. There are two possibilities for the role of HpDML1 in DprA-DNA complex formation. **Model A: C-C interaction plays role in intra-nucleoprotein complex formation.** DprA forms dimer using N-N interaction. These dimers bind to ss and ds DNA in a sequence independent manner. Two such dimers of HpDprA interact with each other using C-C interaction and thus form a continuous nucleoprotein filament on single DNA molecule (Fig 7A). **Model B: C-C interaction plays role in inter-nucleoprotein complex formation.** Another possibility is that HpRF forms dimers using hydrophobic interactions and intramolecular hydrogen bonding as described by Wang et al. (2014) but uses some other motif to mediate interaction between two dimers thus, forming a nucleoprotein filament. Now two such filaments may interact with each other using C-C interaction forming higher order polymeric complex (Fig 7C). It has been shown previously that HpDprA protects DNA from nucleases by completely coating it and thus preventing the access of various nucleases to DNA [16]. If the ‘model A’ is true, deletion of HpDML1 will result in a discontinuous nucleoprotein filament (Fig 7B). Such a nucleoprotein complex will be less efficient in protecting the bound DNA from the nuclease (Fig 7B). If the C-C interaction is involved in inter- nucleoprotein filament interaction then the removal of HpDML1 will result in isolated DNA protein filaments but the integrity of single nucleoprotein filament will not be compromised (Fig 7D). Hence, treatment with nuclease will not be able to discriminate between HpRF-DNA complex and DprA-DNA complex if the ‘model B’ is true. HpRF is able to offer equally efficient protection from nuclease to ssDNA and dsDNA (Fig 4) showing that HpRF alone is sufficient to completely coat single molecule DNA. AFM images confirm the difference in binding pattern of HpDprA full length protein and HpRF. As can be seen in Fig 5C, HpRF binds a DNA molecule by entirely occupying all the available space but forms nucleoprotein filaments isolated from each other. In contrast to full length HpDprA, which forms tightly packed, condensed, extensively cross linked poly-nucleoprotein complexes (Fig 5B), HpRF forms much thinner complexes with DNA. In the electron micrographs of SpDprA–DNA complex, extensive cross filament interaction was observed resulting in a dense molecular aggregate (15). Similar kinds of complexes with DNA were also observed for *Bacillus subtilis* DprA under AFM(17). Thus, we propose that HpDprA binds to a single DNA molecule (single strand or double strand) mainly as a dimer formed through N-N interaction. Such multiple individual nucleoprotein filaments come together and interact with each other through C- C interactions resulting in dense and intricate poly–nucleoprotein complex.

Mutation of the residues shown to be involved in binding ssDNA from crystallographic data resulted in reduced binding affinity with dsDNA as well. The fold reduction in binding affinity of dsDNA was lower than that for ssDNA. Nevertheless it is obvious that the same positively charged pocket which is primarily involved in ssDNA interaction could be responsible (at least partially) for binding with dsDNA. However, the residues crucial for interaction with ss and dsDNA may be different. In conclusion, we have observed a novel site of oligomerization for HpDprA which elucidates the role of C-C interaction in inter-nucleoprotein filament interaction. It would be interesting to further investigatethe effects of HpDML1 deletion on the transformation efficiency of *H*. *pylori* to understand these mechanisms better.

## Supporting Information

S1 File (PDF)
