# Supplementary material for: Insights into the Functional Roles of N-Terminal and C-Terminal Domains of Helicobacter pylori DprA
Source: PLoS One. 2015 Jul 2;10(7):e0131116. doi: 10.1371/journal.pone.0131116 (PMC4489622; doi:10.1371/journal.pone.0131116)
Supplement: S1 File — (PDF) [file pone.0131116.s001.pdf]

Fig. A: Purification and mass characterization of HpRF and HpDML1 (A) 0.1% SDS – 10% PAGE of purified recombinant HpRF - $(His)_6$. M: molecular mass standards. Lane 1: Purified HpRF- $(His)_6$ protein. **(B)** 0.1% SDS – 10% PAGE of purified recombinant HpDML1 - $(His)_6$. M: molecular mass standards. Lane 1: Purified HpDML1- $(His)_6$ protein. **(C)** Mass spectra of purified HpRF - $(His)_6$. Three sharp peaks corresponding to molecular mass of HpRF – $(His)_6$ (1) 26.5 kDa (monocharged species) (2) 13.27 kDa (dicharged species) and (3) 8.85 kDa (tricharged species) are obtained **(D)** Mass spectra of purified HpDML1. A sharp peak corresponding to molecular mass of HpDML1 – $(His)_6$ (1) 9.589 kDa is obtained. **(E)** Circular dichroism spectra of HpDprA, HpRF and HpDML1. Full length HpDprA, HpRF or HpDML1 (100 μg/ ml) were used to record the spectra in 1X PBS buffer. The spectra were recorded in the wavelength range 190 - 300 nm in a Jasco J- 500A spectropolarimeter. A subset of spectra for wavelength range 200 nm to 300 nm is shown here. All measurements were recorded at 25˚C. Spectra presented are representative of three independent experiments.

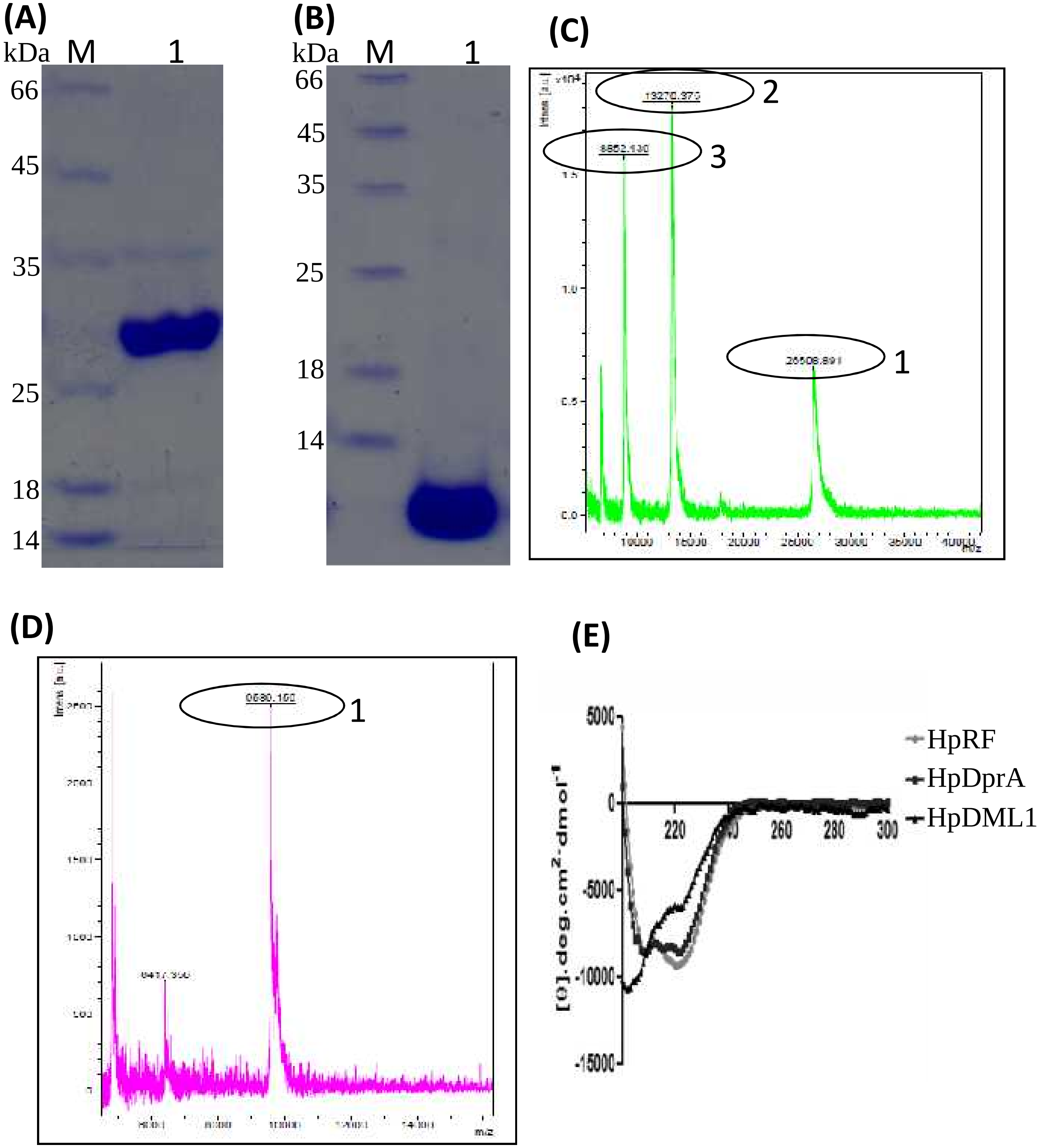
(A)
kDa
M
1
66
45
35
25
18
14
(B)
kDa
M
1
66
45
35
25
18
14
(C)
2
3
1
10000
(D)
1
(E)
5000
0
220
260
280
300
-5000
-10000
-15000
HpRF
HpDprA
HpDML1

Fig. 1

Fig. B: Crosslinking of HpDprA, HpRF and HpDML1 using gluteradehyde. 5 μM of HpDprA **(A)** or 6 μM HpRF **(B)** was incubated with (lanes 2 to 5: (left to right) 0.01, 0.05, 0.1, 0.5%) gluteraldehyde at 4˚C for 10 minutes. **(C)** 140 μM of HpDML1 was incubated with (lanes 2 to 5: (left to right) 0.1, 0.5, 1, 2%) gluteraldehyde at 4˚C for 30 minutes. Lane 1 is protein alone. Lane 6 shows standard protein marker. 1.5 μg of protein was loaded per well. Reaction mixtures were analyzed by SDS-PAGE and visualized by silver staining.

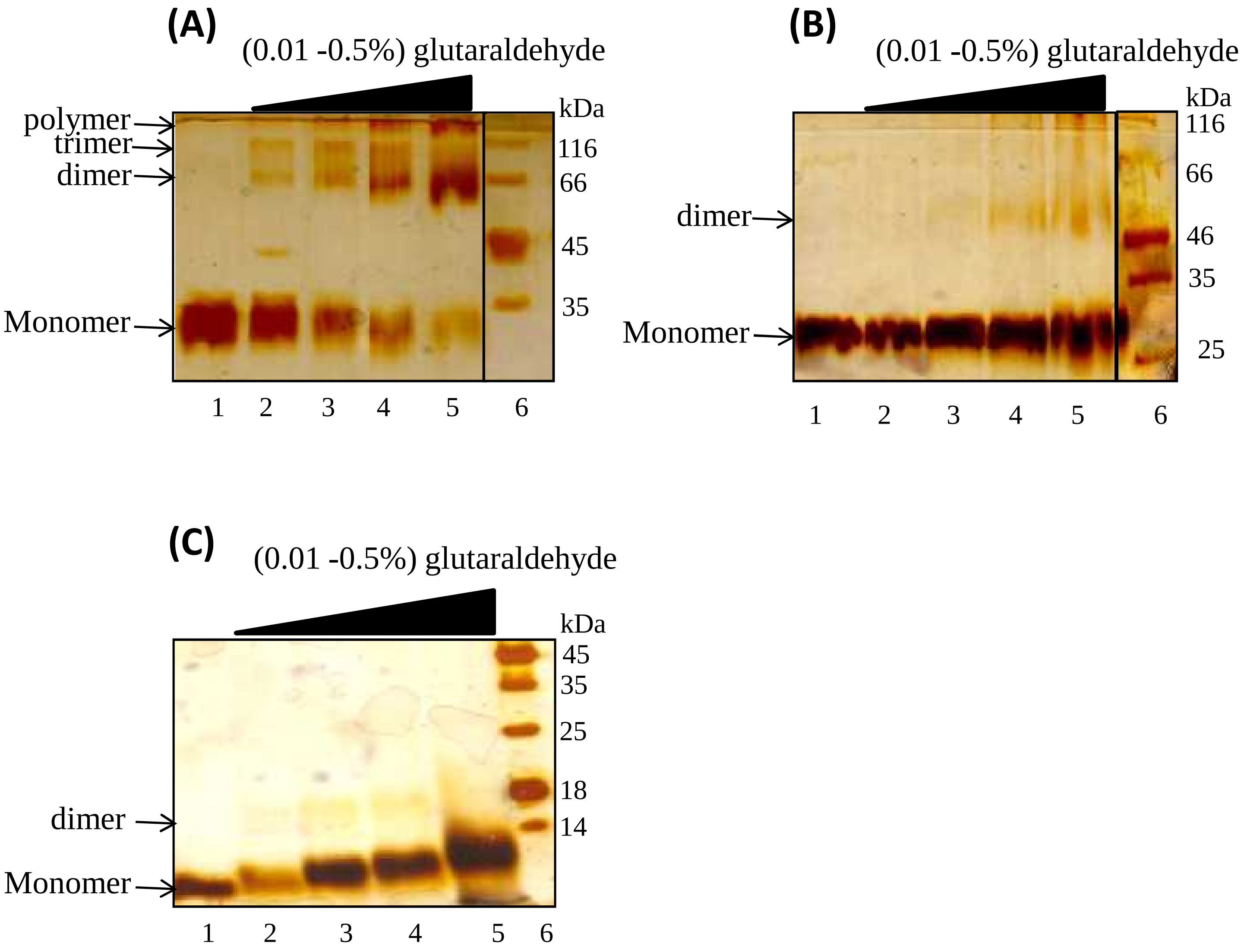
(A)
(0.01 -0.5%) glutaraldehyde
kDa
polymer
trimer
dimer
Monomer
116
66
45
35
1 2 3 4 5 6
(B)
(0.01 -0.5%) glutaraldehyde
kDa
116
66
46
35
25
dimer
Monomer
1 2 3 4 5 6
(C)
(0.01 -0.5%) glutaraldehyde
kDa
45
35
25
18
14
dimer
Monomer
1 2 3 4 5 6

Fig. 2

Fig. C: Purification and CD spectroscopy of HpDprA$_{R48A/R49A}$ and HpDprA$_{R48A/R49A/K133A}$
(A) 0.1% SDS – 10% PAGE analysis of purified recombinant wtDprA and mutants. M: molecular mass standards. Lane1: wild type, lane2: HpDprA$_{R48A/R49A}$, lane3: HpDprA$_{R48A/R49A/K133A}$ **(B)** An overlay of Far – UV circular dichroism spectra of HpDprA, double and triple mutants. The spectra were taken in phophate buffered saline in a wavelength range 190 -300 nm. A subset of spectra from 200 to 250 nm wavelength range is shown here. The observed two minima at 222 nm and 209 nm are characteristic of α – helix spectrum.

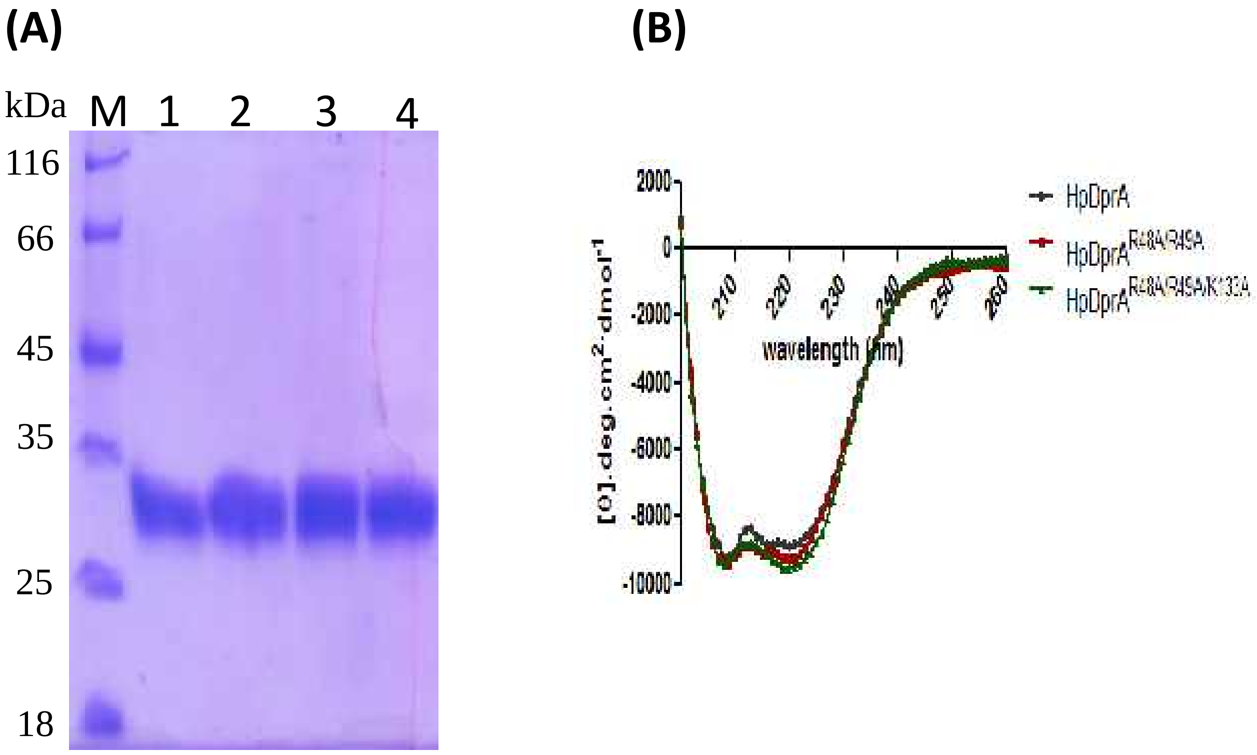
(A)
kDa
M
1
2
3
4
116
66
45
35
25
18
(B)
HpDprA
HpDprA R48A/R49A
HpDprA R48A/R49A/K133A
2000
0
-2000
-4000
-6000
-8000
-10000
210
220
230
240
250
260
wavelength (nm)
[Θ].deg.cm2·dmol-1

Fig. 3
